# Assessing single-stranded oligonucleotide drug-induced effects *in vitro* reveals key risk factors for thrombocytopenia

**DOI:** 10.1371/journal.pone.0187574

**Published:** 2017-11-06

**Authors:** Sabine Sewing, Adrian B. Roth, Michael Winter, Andreas Dieckmann, Cristina Bertinetti-Lapatki, Yann Tessier, Claudia McGinnis, Sylwia Huber, Erich Koller, Corinne Ploix, John C. Reed, Thomas Singer, Andreas Rothfuss

**Affiliations:** 1 Roche Pharma Research and Early Development, Roche Innovation Center Basel, Basel, Switzerland; 2 Roche Pharma Research and Early Development, Roche Innovation Center Copenhagen A/S, Hørsholm, Denmark; Ludwig-Maximilians-Universitat Munchen, GERMANY

## Abstract

Single-stranded oligonucleotides (ON) comprise a promising therapeutic platform that enables selective modulation of currently undruggable targets. The development of novel ON drug candidates has demonstrated excellent efficacy, but in certain cases also some safety liabilities were reported. Among them are events of thrombocytopenia, which have recently been evident in late stage trials with ON drugs. The underlying mechanisms are poorly understood and the risk for ON candidates causing such events cannot be sufficiently assessed pre-clinically. We investigated potential thrombocytopenia risk factors of ONs and implemented a set of *in vitro* assays to assess these risks. Our findings support previous observations that phosphorothioate (PS)-ONs can bind to platelet proteins such as platelet collagen receptor glycoprotein VI (GPVI) and activate human platelets *in vitro* to various extents. We also show that these PS-ONs can bind to platelet factor 4 (PF4). Binding to platelet proteins and subsequent activation correlates with ON length and connected to this, the number of PS in the backbone of the molecule. Moreover, we demonstrate that locked nucleic acid (LNA) ribosyl modifications in the wings of the PS-ONs strongly suppress binding to GPVI and PF4, paralleled by markedly reduced platelet activation. In addition, we provide evidence that PS-ONs do not directly affect hematopoietic cell differentiation in culture but at higher concentrations show a pro-inflammatory potential, which might contribute to platelet activation. Overall, our data confirm that certain molecular attributes of ONs are associated with a higher risk for thrombocytopenia. We propose that applying the *in vitro* assays discussed here during the lead optimization phase may aid in deprioritizing ONs with a potential to induce thrombocytopenia.

## Introduction

Oligonucleotide-based therapeutics constitute a promising drug modality to treat diseases in a gene-specific manner. While generally well tolerated, these single-stranded oligonucleotides (ONs) that hybridize with cellular RNA targets sometimes are associated with clinical adverse effects including hepatotoxicity, kidney tubular toxicity or pro-inflammatory effects (injection site reactions and flu-like symptoms) [1–6]. The mechanism of these adverse effects is not fully understood, but mechanisms involving hybridization to off-target RNA sequences [2] or aptameric binding to proteins [7, 8] have been shown to be contributing factors.

Recently, cases of severe thrombocytopenia were reported in two phase 3 trials with 2’-O-methoxyethyl (2’-MOE)-modified phosphorothioate oligonucleotides, IONIS-TTR_RX_ and volanesorsen. These findings triggered an investigation of a clinical safety database of over 2,600 subjects treated with 16 different MOE-ONs [9]. The analysis—which excluded data from oncology trials as well as the IONIS-TTR_RX_ and volanesorsen trials—concluded that no generic class effect on platelet numbers could be observed and no platelet levels below 50 K/μl were seen in any of the investigated trials. Although the etiology of the unexpected severe thrombocytopenia remains to be elucidated, the clinical safety database analysis offers important insights into MOE-PS-ON-induced platelet effects. Of the 2600 subjects, 0.3% had mild platelet reductions resulting in post-baseline platelet count between 100 and 75 K/μl. A trial comparing patients that were on antithrombotic and/or antiplatelet concomitant medications did not show an increase in bleeding or a change in platelet function. For three of sixteen MOE-PS-ONs, platelet declines of > 30% incidences were observed, suggesting that this finding may be sequence-dependent. In addition, these effects appeared to be time- and dose-dependent, starting at doses higher than 175 mg/week and requiring more than one month of dosing. Importantly, no effect on bleeding was observed with MOE-PS-ONs.

ON-associated decrease of platelet counts is a commonly observed toxicity during nonclinical safety assessment of ONs with various manifestations across species [10–12]. In mice, thrombocytopenia (30–50% platelet decrease) was frequently reported with first generation (fully phosphorothioated without sugar modifications) ONs where it was associated with splenomegaly subsequent to pro-inflammatory effects [10]. Consistent with this notion, the incidence and severity of thrombocytopenia has generally decreased with the modification of ON chemistry to reduce their inflammatory propensity. Published data suggest that thrombocytopenia occurs less frequently in monkeys, where it appears to be associated with high ON dose levels (20 mg/kg/wk or higher) and individual ON sequences, rather than indicating a “class effect” [12, 13].

Intriguingly, besides the above mentioned dose-dependent effects on platelets, which are usually mild to moderate in severity, recent published experience from monkey toxicity studies show that severe reductions in platelet counts (< 75k) can be occasionally seen in individual animals. As outlined by Frazier [12], these findings occur at a low incidence with no clear dose-dependency. Reductions in platelet counts can occur as late as after 6–13 weeks of dosing, are reversible upon dosing cessation and re-appear following re-exposure, suggesting an immune-mediated mechanism.

Given the accumulating data of preclinical and clinical thrombocytopenia, investigations of the mechanisms underlying the observed platelet reductions are clearly warranted. Recent work by Flierl et al. [14] has provided initial evidence that physicochemical properties of ONs associated with their phosphorothioate (PS) backbone modification are a key factor contributing to platelet activation. The authors could show that PS-modified ONs are able to bind to human platelets, possibly via the platelet-specific receptor glycoprotein VI (GPVI), resulting in platelet activation and aggregation [14]. In another study, Jaax et al. [15] hypothesized that negatively charged nucleic acids can bind to platelet factor 4 (PF4), leading to a conformational change of the protein similar to that induced by heparin and known to be the underlying mechanism of heparin-induced thrombocytopenia (HIT). As expected, human heparin-PF4 antibodies cross-reacted with nucleic acid-PF4 complexes, and the antigen-IgG complexes were capable of initiating platelet activation, similar to what is seen in HIT.

Due to their polyanionic nature, PS ONs have a moderate to high binding affinity to various proteins including serum proteins, heparin-binding molecules and several growth factors [7, 16]. New generation ONs are not only PS-modified but also contain building blocks with sugar modifications at the 2’-position including 2’-O-methyl (2’OMe), 2’-O-methoxyethyl (MOE), locked nucleic acid (LNA) or constrained ethyl (cEt) substitutions. ONs supporting RNase H activity routinely contain several of these modifications at the wings which further increase resistance against nucleolytic degradation and also increase binding affinity to their RNA targets [17]. In addition, the introduction of these modifications has been demonstrated to reduce some of the toxic liabilities observed with PS-modified ONs [18, 19].

In this work, we investigated whether the observations by Flierl et al. [14] can be extended to LNA-modified ONs. Moreover, we investigated in a systematic manner potential *in vitro* thrombocytopenia risk factors. Our findings support previous observations that PS-ONs can bind to and activate platelets and we demonstrate that binding and activation correlates with ON length and PS-load. Introduction of LNA modifications in the wings of the PS-ONs strongly suppresses binding to GPVI and PF4 paralleled by markedly reduced platelet activation. In addition, we provide evidence that PS-ONs do not directly affect platelet lineages and differentiation. Furthermore, some ONs used in this study showed a (length-independent) pro-inflammatory potential, which might contribute to platelet activation. Of interest, the pro-inflammatory potential was strongly suppressed when the PS-ONs were modified with LNAs. Overall, our data confirm that certain molecular attributes are associated with a higher thrombocytopenic potential, which we propose can be addressed *in vitro* using appropriate preclinical assays when selecting new ON drug candidates.

## Materials and methods

### Ethics statement

The use and analysis of human whole blood and platelets for this study was approved by the responsible ethics committee (Ethics committee Northwest/Central Switzerland EKNZ/Ethikkommission Nordwest- und Zentralschweiz) before start. Human whole blood was drawn from healthy volunteers after obtaining written informed consent in compliance with the Swiss Human Research Law (Human Research Act (HRA)/ Humanforschungsgesetz (HFG)) and Roche guidelines (Roche Corporate Policy for Human Specimen and Sample Management).

### Preparation of washed platelets

Blood from healthy volunteer donors was collected and anticoagulated in sodium citrate containing vacutainers (BD Vacutainer®, #367714) and then centrifuged at 200 x g for 10 minutes at room temperature (RT). Platelet rich plasma (PRP) was taken from the upper 2/3 of the supernatant to avoid contamination from the buffy coat layer. Washed platelets were subsequently prepared by two additional washing steps at 500x g for 10 min with ACD buffer (acid citrate dextrose; 39 mM citric acid, 75 mM sodium citrate, 135 mM dextrose, 0.01 U/ml Apyrase, pH 7.4). Platelet counts were determined on a Sysmex XT hematology analyzer (Sysmex, Kobe, Japan) and adjusted to a final cell count of 1–3 x 10^8^ platelets/ml in Tyrode’s buffer (137 mM NaCl, 2.68 mM KCl, 11 mM NaHC03, 10 mM Hepes, 0.42 mM Na2HPO4, 5 mM Glucose, 1 mM MgCl2, 2 mM CaCl2, 0.35% BSA, pH 7.4).

### Platelet activation assay

An aliquot of 90 μl washed platelets was incubated with 10 μl of 100 μM ON test preparations or 200 μM ADP or TRAP agonists used as positive controls and incubated for 10 min at RT without agitation. An antibody cocktail of 20 μl CD61 (PE mouse anti-human CD61; BD# 555754) as gating antibody combined with 20 μl PAC1 (FITC anti-GPIIb/IIIa PAC-1; BD, # 340507), and 20 μl CD62P (APC mouse anti-human CD62P; BD, #550888) for the detection of activated platelets was added to the sample, incubated for 20 minutes at RT and then fixed with 200 μl cell fix buffer (BD # 340181) for subsequent flow cytometry evaluation. Each of the preparation was done on triplicates.

### Flow cytometry

Platelet activation was determined by the increase in mean fluorescence binding intensity (MFI) of the activation marker P-selectin (CD62P) and PAC-1 (activated GPIIb/IIIa) on CD61 gated washed human platelets on a Gallios Flow Cytometer by means of the Kaluza Acquisition Software (Beckman Coulter Life Sciences, Indianapolis USA) on least 50’000 CD61^+^ events. Cutoff levels on unstimulated platelets were determined by a 95% confidence interval and results presented in box and whisker plot using GrapPad Prism V 6.07 (GraphPad Software, Inc. La Jolla, CA 92037 USA).

### PF4/heparin ELISA

The assay was run according to the protocol from Petrucci and co-workers [20] with slight modifications. All the steps were conducted at RT. Briefly, microtiter plates (Maxisorp 96well, Nunc #442404) were coated with human recombinant PF4 (10 μg/ml, ProSpec-Tany TechnoGene, Rehovot, Israel) in phosphate buffered saline (PBS) for 2h followed by the addition of either vehicle, heparin (0.002–200 μg/ml, Sigma-Aldrich, Darmstadt, Germany) or ONs (0.001–3 μM). After an overnight incubation, the plates were washed 3x with 0.1% Tween-20 and blocked with 3% BSA in PBS for 2 h. After washing with 0.1% Tween-20 plates were incubated with KKO antibody (0.1 μg/ml in 0.005% Tween 20, ThermoFisher Scientific, Waltham, MA, USA) for 1h. The plates were washed with 0.1% Tween-20 and further incubated for 1 h with the HRP conjugated goat anti-mouse IgG antibody (1:3000 dilution in 0.005% Tween-20, Bethyl Laboratories). Plates were washed with 0.1% Tween-20 and incubated with the HRP substrate BM Blue POD (100 μl/well, Roche Diagnostics GmbH) and further incubated until color development (10–30 min). The reaction was stopped by the addition of stop solution (1N H_2_SO_4_) and absorbance was detected at 450nm on a plate reader (EnSpire, Perkin Elmer).

### GPVI binding assay using surface plasmon resonance

Platelet recombinant human GPVI Protein (aa21-267) with a C-terminal His_6_ tag (Mr 27.7kDa) was obtained from R&D systems (Minneapolis, MN, USA). Protein was dissolved in PBS buffer to a concentration of 100μg/ml. Bovine Collagen Type I with heterogeneous mass ranging between 22 and 110kDa was obtained from Thermo Fisher Scientific (Waltham, MA, USA).

All SPR experiments were performed on the Biacore® T200 (GE Healthcare, Uppsala, Sweden) instruments at 18°C in the running buffer composed of 10 mM phosphate buffer, 2.7 mM KCl 137mM NaCl, pH 7.4 at flow of 5 μl/min or 30 μl/min.

Immobilization of capturing anti-His antibody (His capture kit, 28-9950-56, GE Healthcare, Uppsala, Sweden) was performed in running buffer containing 10 mM phosphate buffer, 2.7 mM KCl, 137mM NaCl, pH7.4. First CM5 sensor surface was activated 10 min with a solution of 0.2 M N*-*ethyl-N-dimethylaminopolycarboodiimide (EDC) and 0.05 M N-hydroxysuccinimide (NHS). After activation, the sensor surface was contacted with the anti-His antibody (50 μg/ml in 10mM sodium acetate buffer, pH 4.5) to reach immobilization level of ~2000 and 6000 resonance units (RU) on various channels. Excess of activated carboxylic groups on the sensor surface was quenched with ethanolamine (1 M, pH 8.5, 7 min). GPVI protein was captured on anti-His capturing antibody. Here the sensor was conditioned with 3 consecutive 1min injections of 10 mM glycine-HCl, pH 1.5. Next, GPVI protein was diluted in running buffer to 5.5μg/ml and applied over anti- His Ab sensor surface to achieve immobilization levels of GPVI of about 150–200 or 650–1000 RU. ONs were diluted in running buffer to the final test concentrations as indicated in the figures and tested in replicates. After each injection of ON the complex of GPVI and ON was washed off with short pulses of 10 mM glycine-HCl, pH 1.5 and subsequently GPVI was captured again. Bovine collagen type I was used as a positive control to follow the protein stability over time.

SPR data analysis was performed using Biacore T200 Evaluation Software (version 2.0) and Excel (Microsoft Office 2010, version 14.0). All binding resonance signals were single referenced, i. e. signals monitored on the binding active channel were subtracted with signals from reference channel (sensor surface modified with anti-His Ab). All data were normalized by the capturing level and molecular mass. Stability points monitored 10 sec after the end of the association phase in the binding sensorgram were evaluated to rank ONs.

### Human bone marrow toxicity assay

Human mononucleated cells (hMNCs) were received cryopreserved from AllCells, LLC or STEMCELL Technologies Inc, and were isolated from bone marrow of healthy volunteers. Bone marrow cells and tissue were drawn from the posterior ilia crest and subsequently purified by density gradient separation and lysis of red blood cells, prior to cryopreservation. Cryopreserved cells were at least 90% viable after thawing according to the Certificate of Analysis.

On the day of experiment, hMNCs were thawed rapidly using DNase to avoid cell clumping and then washed twice in IMDM medium supplemented with 10% FCS to a final cell density of 3.3x10^6^ cells/ml. Test substances were received as 10 mM stocks dissolved in PBS and a concentration range (5–6 concentrations, in 1:2 dilution steps) was prepared by diluting this stock solution into 100% PBS. This concentration range was then diluted directly 1:10 in cell suspension. This protocol resulted in solvent concentration of 10% PBS final assay concentration.

The hematopoietic cell lineage-specific medium for cultivation of bone marrow *in vitro* was purchased from Hemogenix, Inc (Cat# SEC-HPP2-40H; SEC-MK1-40H). HPP2 medium supports the culture of primitive, expanding lympho-hematopoietic stem cells as well as progenitor cell populations and contains the growth factors EPO, GM-CSF, IL-2, IL-3, IL6, IL7, SCF, TPO and Flt3-L. MK1 medium supports the megakaryocyte lineage and contains the growth factors IL-3, TPO and Flt3-L.The exact composition of the medium is proprietary to Hemogenix.

Pre-dilution of cell suspension into the hematopoietic cell lineage specific medium was performed using a 1:10 dilution step. For each lineage-specific incubation, 22.5 μl of medium suspension (containing 7500 cells for hBM MNCs) were seeded per well of a 384-well plate, and incubated for 3 hours after seeding. ON treatment was initiated 3 hours after seeding. Four replicates of each concentration range were prepared. As solvent controls, incubations in 10% PBS were included. Cells were incubated at 37°C and 5% CO_2_ for 5 days.

Endpoint of the assay after incubation was intracellular ATP (iATP) measurement, using the LiveGlo assay kit from HemoGenix. A volume of 25 μl of ATP reagent was added to each well of the 384 well assay plate and the plate left for 10 min at room temperature. The resulting luminescence signal was recorded on a Synergy H1 multi-mode Reader (BioTek Instruments Inc.), and results converted into iATP using a calibration curve of known ATP concentrations.

### Immunotoxicity assays

Venous blood from healthy donors was collected in anticoagulant-sprayed vacutainer tubes (Roche medical Center, Basel, Switzerland) and kept at room temperature. Within 3 hour of collection, 195 μl of fresh whole blood was added in triplicates to U-bottom wells of 96-well plates containing 5 μl of 40 fold concentrated ON stock solution. These conditions ensure optimal accomplishment with respect to practicability and efficiency to gain at least 80 μl of plasma to conduct multiple Complement split fragment, cytokine and chemokine ELISA analyses. Internal references for complement stimulation were also included (Complement activator HAGG Heat Aggregated Gamma Globulin, TECOmedical and Zymosan, Sigma). Blood cells responsiveness to innate immune stimuli and endogenous activation level were assessed by including controls containing PBS, stabilizing solution, and TLR activators (R848, CpG and polyDC, invivoGen).

After incubation at 37°C with 5% CO2, cells and plasma were separated by centrifugation at 1800g for 5 min. Plasma samples were collected at 6 hours stored at -80°C until cytokine and chemokine analysis. Plasma samples for Complement analysis were collected after 45 min, diluted 1:1 in stabilizing solution and frozen in aliquots at -70°C until ELISA.

Determination of cytokine concentrations was performed by ELISA using the Human Cytokine chemiluminescent assay kit (Aushon SearchLight, Cat. No 101-261-1-AB) and Human MCP-1 Array (Aushon SearchLight, Cat. No 51-100-1-AB) with the SignaturePLUS™ imaging system and the PROarray analysis software. Reconstitution volume of IL-6 and IL-8 standards was adapted to extend the standard curve up to 2000pg/ml and 8000pg/ml respectively. Lower limit of quantification (LOQ) was measured at 0.01 pg/ml for IL-6 and IL-8, 0.11 for IFNγ and 0.02 pg/ml for TNFα.

For determination of complement fragment release, plasma samples were thawed and diluted in the sample diluent according to manufacturer’s instructions. Analyte concentrations were determined by individual sandwich ELISA using the different human assay kits according to the manufacturer's instructions (Human Complement Plus EIA kits: C3a Cat No. A015, C5a Cat No. A025, from Quidel TECOmedical AG, Sissach, Switzerland). Plates were analyzed on Versamax microplate ELISA reader (Bucher Biotec, Basel, Switzerland) using the Softmax Pro software v.5.2.

Triplicate values were averaged for each concentrations and stimulation index (SI, relative to PBS or scrambled ON) was calculated for each donor. Mean stimulation index for the 3 donors was used to rank the immunotoxicity potential of the test items.

### Oligonucleotides

We designed a set of PS-modified tool ONs which allowed us to investigate the effects of varying oligonucleotide length, PS content and LNA wing modifications on platelets independent of the ON sequence. The PS-ONs consisted of increasing numbers (*n* = 5–11) of AC dinucleotide-repeats (AC)*n*, and, where indicated, had three flanking LNA modifications on each side. In addition, we synthesized, ODN2395 Thio (+PS backbone) and ODN2395 (-PS backbone) [14] as assay controls.

All tested molecules were 10–22 nucleotide long single-stranded oligodeoxynucleotides (ON), which were all fully phosphorothiotated (PS) except ODN2395 (phosphodiester linkages) and had either a gapmer design with LNA-modified wings or no sugar modification. ONs were solubilized in PBS (pH 7.4) to a stock concentration of 10 mM.

## Results

To understand the potential peripheral and central effects of ONs on platelets we established a battery of *in vitro* assays (Table 1). We investigated: (1) the effect of ONs on platelet activation, (2) their direct interaction with platelet factor 4 (PF4) and platelet collagen receptor glycoprotein VI (GPVI), (3) their bone marrow toxicity in hematopoietic stem and progenitor lineages and (4) their pro-inflammatory potential in human whole blood. Tool molecules with increasing length and varying wing modifications were used to assess effects in the *in vitro* assays, in addition to two ONs described by Flierl et al. [14], ODN2395 Thio (+PS backbone) and ODN2395 (-PS backbone) as assay controls (Table 2).

**Table 1:** *In vitro* assays used for the assessment of thrombocytopenia risk of ONs.

Assay	Readout	Question Addressed
**Platelet Activation**	Surface Activation Markers by FACS	Direct Effect on Platelets
**Binding to Platelet collagen receptor GPVI**	Biacore using recombinant Protein	Platelet Activation via Binding to GPVI
**Binding to Platelet Factor 4 PF4**	ELISA using recombinant Protein	Idiosyncratic Effects via Binding to PF4 and potential Ab Formation (similar to Heparin-induced Thrombocytopenia)
**Bone Marrow Assay**	Viability and differentiation of hematopoietic stem and progenitor lineages	Bone Marrow Suppression as potential contributing MoA
**Immunotox Assay**	Cytokine release and complement activation	Pro-Inflammatory Potential as early Contributor & Marker

**Table 2:** Design of the tool ONs.

Oligonucleotide	Length	Sequence
ODN2395_Thio	22	T*C*G*T*C*G*T*T*T*T*C*G*G*C*G*C*G*C*G*C*C*G
ODN2395	22	TCGTCGTTTTCGGCGCGCGCCG
(AC)5	10	A*C*A*C*A*C*A*C*A*C
(AC)6	12	A*C*A*C*A*C*A*C*A*C*A*C
(AC)7	14	A*C*A*C*A*C*A*C*A*C*A*C*A*C
(AC)8	16	A*C*A*C*A*C*A*C*A*C*A*C*A*C*A*C
(AC)9	18	A*C*A*C*A*C*A*C*A*C*A*C*A*C*A*C*A*C
(AC)10	20	A*C*A*C*A*C*A*C*A*C*A*C*A*C*A*C*A*C*A*C
(AC)11	22	A*C*A*C*A*C*A*C*A*C*A*C*A*C*A*C*A*C*A*C*A*C
(AC)8 LNA	16	A*C*A*C*A*C*A*C*A*C*A*C*A*C*A*C
(AC)9 LNA	18	A*C*A*C*A*C*A*C*A*C*A*C*A*C*A*C*A*C
(AC)10 LNA	20	A*C*A*C*A*C*A*C*A*C*A*C*A*C*A*C*A*C*A*C

Capital letters deoxynucleotides

* phosphothioate backbone; bold letters: locked nucleic acid

### Platelet activation

**Fig 1:**
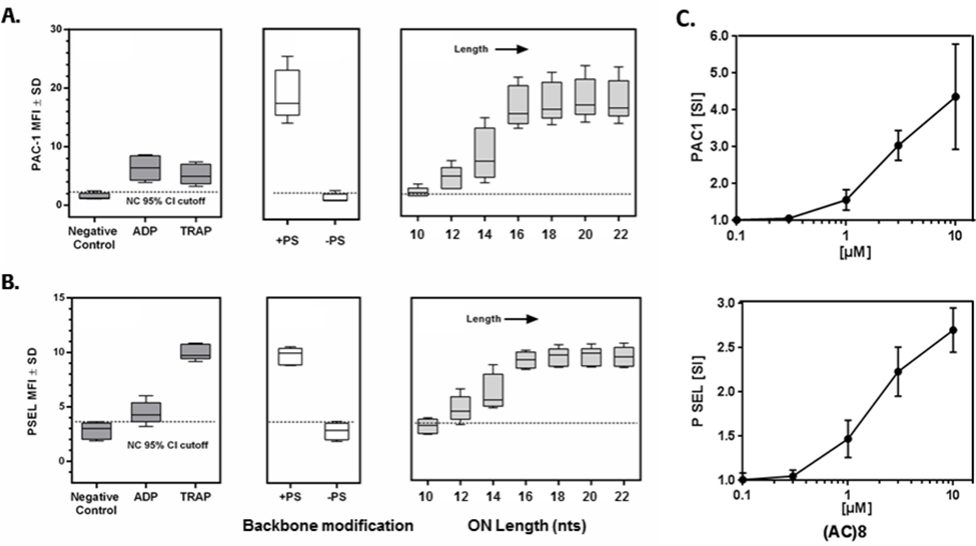
Platelet activation correlates with the number of PS modifications. Increase in platelet activation marker A. PAC-1 (activated glycoprotein IIb/IIIa) and B. P-selectin, after 10 min incubation with indicated controls (20 μM ADP, 20 μM TRAP) or 10 μM ODN2395 Thio (+PS), ODN2395 (-PS) and ONs of different length ((AC)5-11; 10–22 mer). Data are shown as boxplots of n = 5 individual data points showing the median value. Dotted lines represent the 95% ULN confidence interval of unstimulated platelets. C. Concentration dependent effect of (AC)8 on increase in platelet PAC-1 and P-selectin surface expression plotted as stimulation index versus control. Data are means ± SD (n = 3).

The tool ONs were first tested for their ability to activate platelets. The assay control ODN2395 Thio strongly activated human platelets as demonstrated by up-regulation of activated glycoprotein IIb/IIIa (PAC1, Fig 1A) and P-selectin (Fig 1B). We next investigated the contribution of ON length to the ability to activate platelets. We observed a clear increase in the surface expression of PAC1 with increasing length of our (AC)*n* tool ONs, with no activity of the 10-mer (AC)5 and a maximum effect with the 16-mer (AC)8. Further increase in length of the ONs to 18, 20 or 22 nucleotides did not result in higher PAC1 surface expression. Similar results were obtained with P-selectin, another platelet activation marker (Fig 1B). We tested the 16 mer (AC)8 in a concentration response experiment and determined 1 μM as the lowest concentration that results in upregulation of platelet activation markers (Fig 1C).

### GPVI protein binding

To obtain insights into the underlying mechanism of the described platelet activating effect, we determined the binding of the tool ONs to platelet proteins. Flierl et al. [14] have described the involvement of platelet collagen receptor glycoprotein VI (GPVI) in ON-induced platelet activation. Moreover, the authors found that PS-ON molecules shorter than 18 nucleotides did not exhibit a significant platelet-activating effect.

**Fig 2:**
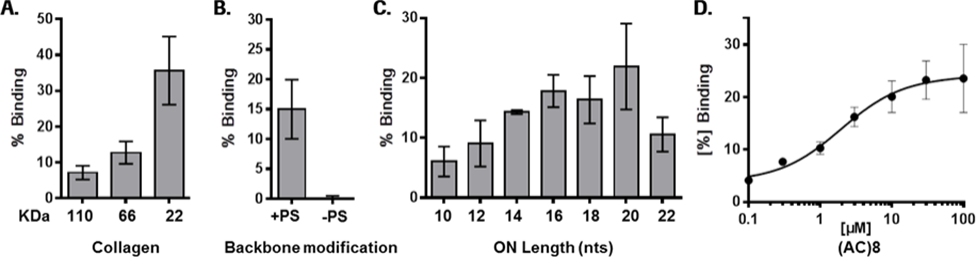
GPVI receptor binding correlates with the number of PS modifications. (A) Binding of bovine collagen type I, (B) ODN2395 Thio (+PS), ODN2395 (-PS) and (C) ONs of different length ((AC)5-11; right) to His-tagged GPVI protein captured on SPR chip via anti-His antibodies. All ONs were tested at 10 μM. Data are means ± SD (n = 24 (A); n = 12 (B)) and means with error bars demonstrating data range from two replicates (C). (D) Concentration response of (AC)8 binding to GPVI. Data are means ± SD (n = 3).

Therefore, we assessed direct interaction of our tool ONs with recombinant human GPVI protein by surface plasmon resonance (Biacore). Since GPVI has increased affinity for collagen as a dimer [21, 22], we used the recombinant extracellular domain of human GPVI (aa21-267) with a C-terminal His tag and immobilized it on a Biacore microfluidic chip via anti-His antibodies to bring two GPVI fragments in close proximity. Bovine collagen type I and the positive control oligonucleotide ODN2395 Thio at 10 μM bound to immobilized GPVI, as expected. No binding was observed with 10 μM ODN2395 lacking thio modifications in the backbone. When the (AC)*n* oligonucleotides were tested at a concentration of 10 μM, a length-dependent increase in binding to GPVI protein was observed with maximal binding of the 20-mer (AC)10 (Fig 2A–2C). In order to determine the concentration dependency of GPVI binding, we run a concentration response binding experiment with (AC)8 and observed half maximal binding at a concentration around 2 μM ON (Fig 2D). Overall, the binding activity of the tested ON tools was similar to that of the collagen control.

### Interaction with PF4

**Fig 3:**
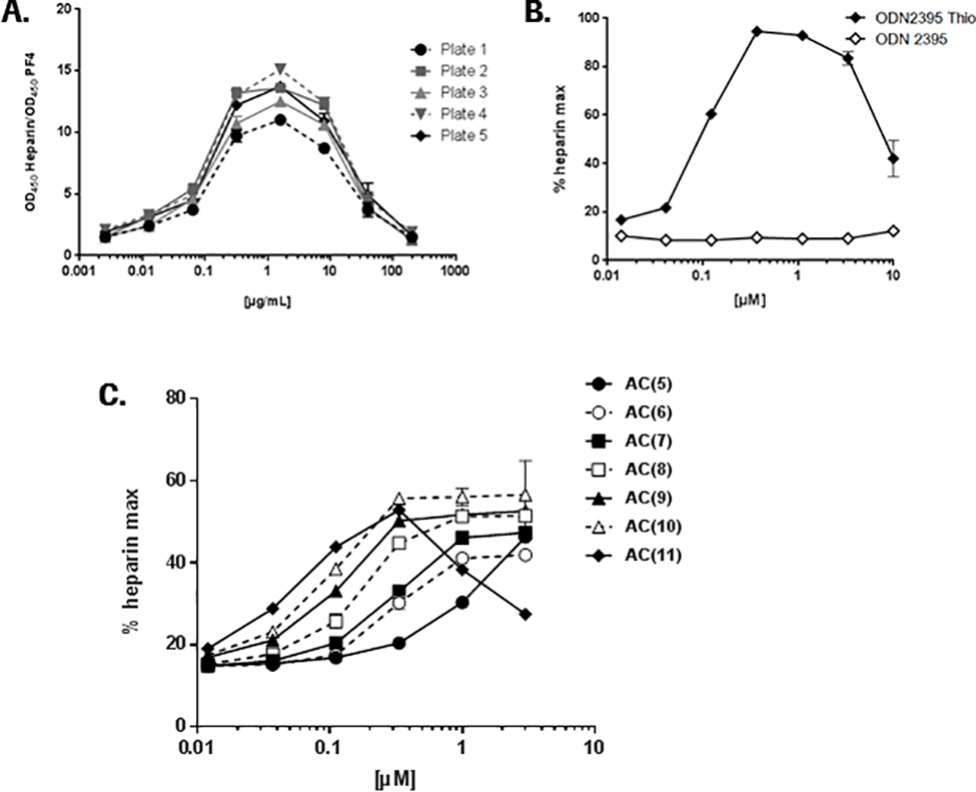
Binding of KKO antibody to PF4-ON complexes. The influence of PS modifications and ON length on PF4-ON complex formation was evaluated by ELISA. (A) Binding of KKO to PF4-heparin was used as a positive control in all experiments. Data from independent experiments (n = 5) are shown. (B) KKO antibody binding to the PF4-ODN2395 Thio complexes and PF4-ODN2395. (C) Binding of KKO to PF4-ON complexes of different length (10mer to 22mer). All data in (B) and (C) are expressed as % maximal heparin binding (% heparin max) and represent mean ± SD (n = 4).

Heparin is known to activate platelets upon repeated administration [23]. It has been shown that the binding of heparin to PF4 induces a conformational change of the protein, leading to the exposure of a neo-epitope that induces the production of anti-PF4/heparin antibodies [24]. To evaluate the property of oligonucleotides to bind and form antigenic complexes with PF4, we used a monoclonal antibody (KKO) that has similar properties to the naturally occurring anti-PF4/heparin antibodies found in patients with heparin-induced thrombocytopenia/thrombosis [25]. The ELISA-based approach showed that the KKO antibody recognizes PF4/heparin complexes in a dose-dependent manner (Fig 3A) following a bell-shaped curve, which is likely due to the recognition of complexes formed at specific molar ratios of the reactants as previously described [26]. The same trend was observed when the tool oligonucleotide ODN2395 Thio was tested in the assay while the oligonucleotide lacking the thio (PS) modification showed no binding (Fig 3B). To assess if the length of ONs has an impact on inducing the conformational change on PF4 to expose the neo-epitope we used our set of (AC)*n* tool compounds. The resulting data revealed a clear dose- and length-dependent activity of ONs in the ELISA assay (Fig 3C). The 22-mer ON (AC)11 showed a bell-shaped curve similar to the heparin control and ODN2395 Thio.

### Bone marrow toxicity

**Fig 4:**
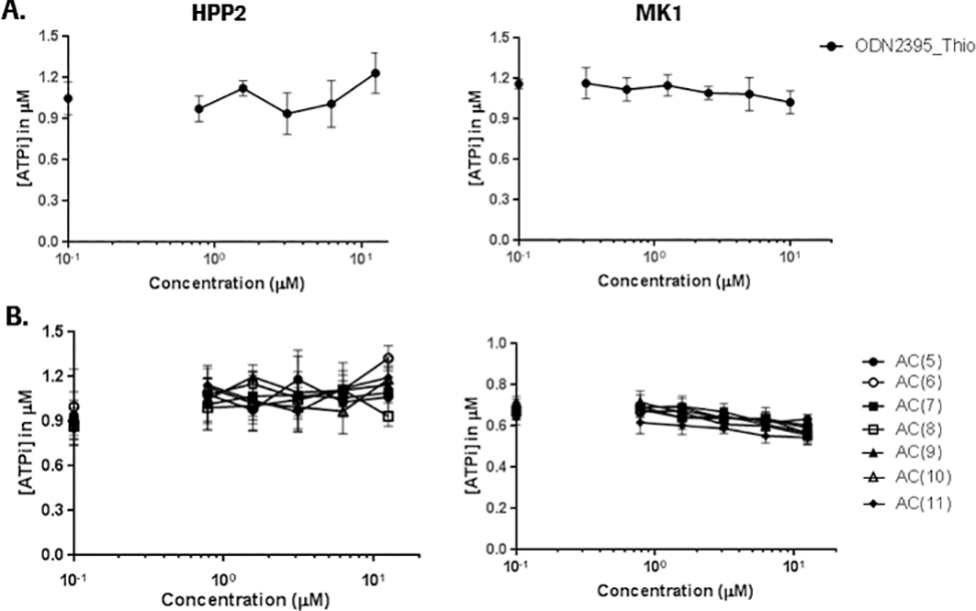
ONs do not show adverse effects on hematopoietic cells. Effects of (A) ODN2395 Thio and (B) ONs (AC)5-11 on ATP content in human primary bone marrow cultured *in vitro* for 5 days in growth factor supplemented medium. HPP2 medium supports proliferation and differentiation of hematopoietic stem and several progenitor lineages. MK1 medium supports proliferation and differentiation of the megakaryocyte lineage. Data represent mean ± SD (n = 4).

Besides a peripheral origin, thrombocytopenia may also originate from a central effect taking place in the bone marrow. To investigate potential adverse effects of ONs on hematopoietic cells, we carried out an *in vitro* bone marrow assays. The assay measures ATP depletion in primary human bone marrow-derived mononucleated cells that are allowed to proliferate and differentiate to early progenitors over a 5-day incubation time in lineage-specific growth factor mixes [27]. For this particular study, two different culture conditions were used: HPP2 medium supports differentiation of lympho-hematopoietic stem cells as well as progenitor cell populations. MK1 medium supports differentiation of the megakaryocyte lineage [28]. No significant effects were observed for any of the ONs tested up to 10 μM under either culture condition (Fig 4), suggesting that test compounds did not exert bone marrow suppression *in vitro*. ODN2395 without thio modification could not be assessed in this assay, due to its instability in our culture conditions in the presence of bone marrow cells (data not shown).

### Impact of sugar (ribosyl) modifications in the wings of ONs

**Fig 5:**
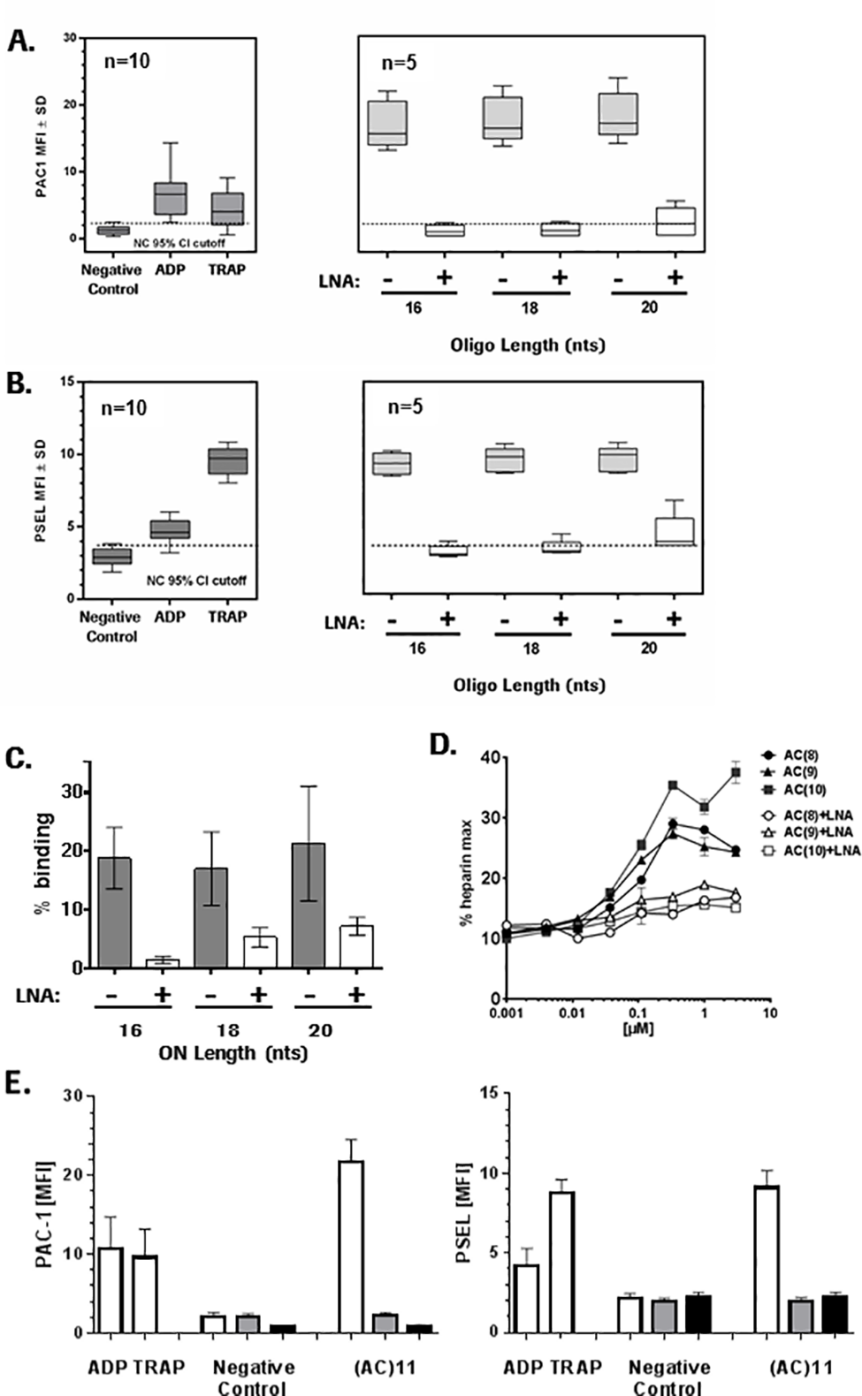
LNA wing modifications of PS-ONs strongly suppress platelet activation and binding to GPVI and PF4. Increase in platelet activation marker (A) PAC-1 (Glycoprotein IIb/IIIa) and (B) P-selectin, after 10 min incubation with indicated controls (20 μM ADP, 20 μM TRAP) or 10 μM ONs with different length (16mer (AC)8, 18 mer (AC)9 and 20 mer (AC)10) without and with 3 LNA modifications in the flanks. (A) and (B) are box-plots of n = 5 or n = 10 individual data points as indicated with the bars showing the median value. Dotted lines represent the 95% ULN confidence interval of unstimulated platelets. (C) Binding ONs of different length ((AC)8-10 without and with 3 LNA modifications in the flanks to His -tagged GPVI protein captured on SPR chip via anti-His antibodies. Data are means with error bars demonstrating data range from two replicates. (D) Activity of ONs of different length ((AC)8-10 without and with 3 LNA modifications in the flanks in the PF4 ELISA expressed as % maximal heparin activity. Data represent means ±SD (n = 4). (E) Changes in platelet activation markers PAC-1 and P-selectin induced by controls (ADP, TRAP and negative control (PBS)) or AC11 (white bars) and in the presence of 5 μM Bay 61–3606 (grey bars) or 5 μM Src inhibitor PP2 (black bars). Data are means ±SD (n = 5).

We investigated the influence of sugar (ribosyl) modifications in our tool ONs on platelet protein binding and platelet activation. Three locked nucleic acids (LNAs) were introduced at the flanking three nucleotides of (AC)8, (AC)9 and (AC)10 (Table 2) then these variants were tested for their ability to activate human platelets and to bind GPVI and PF4. In all three assays the LNA-modified version of the ONs showed strong attenuation of the effects observed with the unmodified molecules (Fig 5). In particular, in the platelet activation assay both PAC1 and P-selectin induction were dramatically reduced by the LNA modifications (compared to the unmodified ONs). Interestingly, a tendency for length dependency of the effects was still evident (Fig 5A and 5B). Similarly, GPVI binding of the LNA versions of (AC)8-10 was significantly reduced compared to the unmodified ON tools, and again a length dependency was seen for the remaining binding activity (Fig 5C). In addition, activity of the LNA molecules in the PF4 ELISA was also strongly attenuated for all three variants, however, in this case no length dependency was seen (Fig 5D).

Finally with the attempt to better understand the pathway leading to upregulation of platelet activation markers by our tool ONs and link that to the GPVI binding data, we investigated the role of Syk1 and Src, two kinases involved in downstream signaling of GPVI, which is a member of the immunoreceptor tyrosine based activation motif (ITAM) receptor family. Incubation of human platelets with the 22 mer (AC)11 ON in the presence of either 5 μM Syc1 kinase inhibitor Bay 61–3606 or 5 μM Src inhibitor PP2 completely abolished upregulation of platelet activation markers PAC-1 and P-Selectin (Fig 5E).

### Immunostimulatory effect of ONs

It is well established that some ONs have a sequence-dependent propensity for activating the innate immunity and/or the alternative complement pathways [13, 29, 30]. Thus, soluble mediators of innate immune activation (cytokine, chemokine and complement split fragments) were measured in plasma of human whole blood samples after incubation with controls, ODN2395 Thio and ODN2395 and the (AC)*n* oligonucleotides with and without LNA modification.

**Fig 6:**
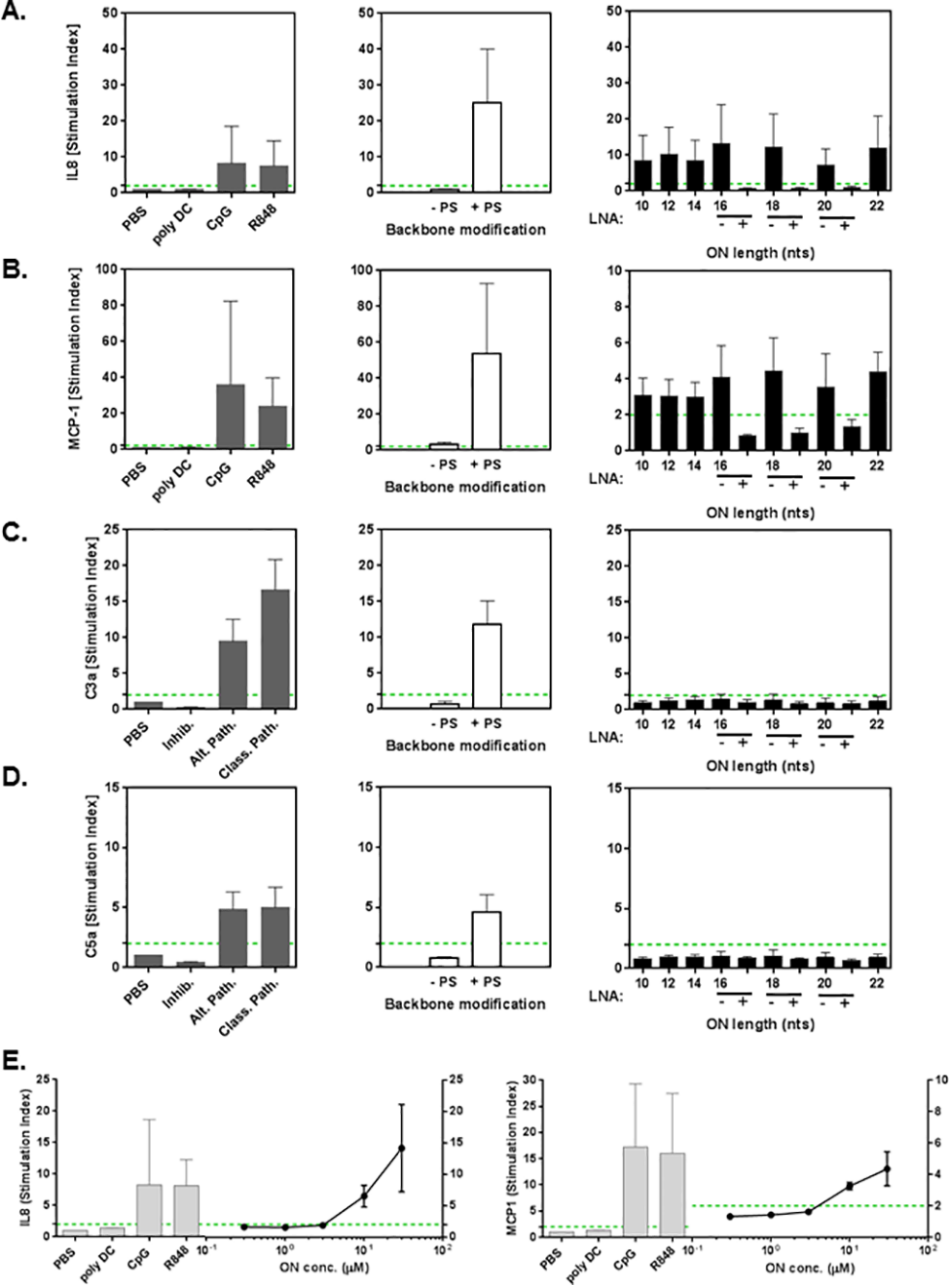
PS-ONs increase cytokine secretion in human blood. (A) IL-8 and (B) MCP-1 increases in human blood after 6 h incubation and with assays controls polyDC, CpG and R848 (left) and with ONs ODN2395 Thio (+PS), ODN2395 (-PS) (middle) and ONs (AC)5-11 (10 to 22 mers) at 50 μM. Unmodified and LNA versions of (AC)8, (AC)9 and (AC10) are labelled (-) and (+), respectively (right). Increases in (C) C3a and (D) C5a in human blood incubated for 45 min with assays controls (alternative pathway stimulated by Zymosan, classical pathway stimulated by Complement activator) with ONs ODN2395 Thio (+PS), ODN2395 (-PS) and ONs (AC)5-11 (10 to 22 mers) at 50 μM. Unmodified and LNA versions of (AC)8, (AC)9 and (AC10) are labelled (-) and (+), respectively. (E) Concentration response of (AC)8 on IL-8 and MCP-1 secretion in human blood. Dotted lines represent the 95% ULN confidence interval of unstimulated platelets. Data represent means ±SD (n = 5).

When tested *in vitro* using human blood-based immunotoxicity screening assays, a strong increase in cytokine secretion was observed in blood incubated with ODN2395 Thio, with >20 fold stimulation of IL-8 and MCP-1, while ODN2395 without PS modification did not result in cytokine release (Fig 6A and 6B). A similar pattern is seen for complement split fragments C3a and C5a (Fig 6C and 6D). For all of the (AC)*n* ONs, when tested at 50 μM (a concentration that is generally far in excess of clinically used doses), we observed an 8–13 fold increase in IL-8 relative to unstimulated whole blood and a three to four fold increase in MCP-1 (Fig 6A and 6B right panel). These increases in cytokine production were length independent and confined to IL-8 and, to a lesser extent, to MCP-1. No effect on complement split factors was seen with any of the length tool ONs (Fig 6C and 6D right panel). Of note, LNA modifications of the (AC)*n* ONs strongly suppressed the effects of the parent ONs on IL-8 and MCP-1 secretion. We finally tested the effect of different concentrations of (AC)8 on IL-8 and MCP-1 release in human blood. No effect was observed at the lower concentrations up to 3 μM of (AC)8, while at 10 μM a clear increase was observed for both cytokines (Fig 6E).

## Discussion

Thrombocytopenia induced by ONs may have several underlying mechanisms. Among the suspected mechanisms are (a) central effects of ONs on platelet production, (b) direct effect of ON by interacting with platelet proteins and subsequent platelet activation and (c) aggregation or an immune-mediated effect similar to what is described for heparin-induced thrombocytopenia, which can be fast or delayed depending on the presence of preexisting antibodies [12, 31, 32].

The work presented here investigated these potential mechanisms *in vitro* and confirmed previous observations made that ONs can cause direct or immune-mediated activation of platelets, which potentially explains the thrombocytopenia observed in some clinical contexts. While the exact underlying mechanism of such events in the clinic remains unknown and may be multifactorial, our data provide further supporting evidence for factors previously suggested to play a role. Among these, the length of a given ON and specifically, the number of PS-backbone modifications introduced into the sequence are suggested to be key drivers increasing the intrinsic risk for binding to platelets leading to their activation (Fig 1). This is in line with recent work by Flierl et al. [14] investigating the impact of PS on binding to platelet proteins including GPVI. Using a series of tool ONs with different lengths and a common sequence, we could clearly demonstrate that also the length of a PS-ON correlates with its binding potential to GPVI (Fig 2) and its potential to activate platelets (Fig 1), while a non-PS ON (ODN2395) was inactive in both assays. The PS-ON length correlation seen for both the binding to GPVI and the activation of platelets supports recent findings that PS-ON-induced platelet activation is mainly mediated via binding to GPVI [14]. In accordance with that, we also demonstrate that upregulation of platelet activation markers is completely abolished by ONs, when we inhibited downstream signaling of GPVI by Syk and Src kinase inhibitors.

Evidence has been presented that oligonucleotides also interact with platelet proteins other than GPVI such as PF4, which is a CXC chemokine family cytokine secreted by activated platelets during platelet aggregation and a known target of heparin leading to HIT [33, 34]. The potential of ONs to induce the generation of drug-PF4 antibodies capable of activating platelets has been shown by Jaax et al. [12, 13, 15]. Our data demonstrating binding of PS-ONs to PF4 support the potential of these molecules to induce a HIT-like idiosyncratic pattern *in vivo* (Fig 3; [34]). Similarly to GPVI, binding of the PS-ONs to PF4 was length-dependent.

In contrast to platelet activation (destruction), we did not detect an impact of ONs on bone marrow suppression *in vitro*, which argues against a direct effect of ONs on platelet maturation (Fig 4). But we cannot completely rule out other possible central mechanisms based on the MK differentiation and growth assay used here. Not covered by this assay were potential effects on late maturation, migration to the vascular niche, or terminal platelet formation and release, all highly dependent on interactions of megakaryocyte membrane receptors (including GPVI) with its microenvironment in the bone marrow.

Since new generation ONs supporting RNase H activity are not only PS-modified but also contain flanking building blocks with sugar modifications at the 2’-position, we also assessed the impact of three LNA modifications in the flanks of our tool ONs on platelet binding and activation. Strikingly, the addition of three LNA modifications into the flanks of the tool PS-ONs dramatically suppressed GPVI and PF4 binding and platelet activation (Fig 5). Of note, except for binding to PF4, a trend for length dependency was still evident for the LNA-modified ONs (Fig 5A–5C).

When added to whole blood at a high concentration (50 μM), all fully PS-modified tool ONs stimulated release of IL-8 and to a lesser extent MCP-1, irrespective of their length. In contrast, no cytokine secretion was seen with LNA-modified ONs. We determined the lowest ON concentration where cytokine release is triggered to be at 10 μM. Generation of complement split fragments C3a and C5a was not stimulated by unmodified or LNA-modified ONs of various lengths but was seen with ODN2395 Thio, a known TLR9-activating oligonucleotide [35]. This observation is in line with previous data demonstrating that ONs containing TLR9-activating CpG motifs can activate complement in human whole blood [35], while ONs without these motifs do not [13]. PS-modified ONs have been documented to stimulate innate immune responses via pattern recognition receptors [29], resulting in cytokine/chemokine release and in splenomegaly in animals. Such systemic inflammation has been suspected as a factor for platelet count reduction, either via platelet sequestration, activation or central inhibition by cytokines [10]. Although the above mechanisms have not been fully proven to our knowledge, a correlation between inflammation and platelet reduction has been observed and is a likely hypothesis for the effects as platelet activation and consumption is frequently observed in various acute tissue injury conditions via release of Platelet-activating factor (PAF) and other proinflammatory cytokines from leukocytes and endothelial cells of the damaged area [36]. Therefore, screening against cytokine release potential may, at least indirectly, reduce the risk for thrombocytopenia.

Immune-mediated thrombocytopenia after heparin therapy has been shown to occur in an abrupt manner between day 5 and 12 of heparin treatment in about 0.5 to 1% of patients receiving heparin. In more than 99.9% of patients IgG antibodies against anti-PF4/polyanion complexes are present, consistent with an immune-mediated mechanism. In this regard, antibodies generated against heparin-containing complexes activate platelets via FcγIIa receptors, leading to secretion of platelet-derived pro-coagulant particles and thereby accelerating the generation of thrombin [33]. Typically, for most patients experiencing HIT, the degree of thrombocytopenia is moderate with platelet counts rarely below 60 K/μl. A subset of HIT patients was recently shown to develop autoimmune thrombocytopenia in the absence of the primary factor, heparin [37]. It remains to be determined whether a similar immune-mediated mechanism accounts for thrombocytopenia observed in some patients exposed to PS-ONs. Prior attempts to detect anti-ON antibodies have generally been negative, but the possibility of induced antibodies that recognize ONs when complexed to particular unknown proteins cannot be excluded. It has been recently reported that 60% of the anti-PF4/heparin antibodies that induced platelet activation in the presence of heparin also caused platelet activation in the presence of nucleic acids [12, 15] which suggests that only some aspects of ON-induced thrombocytopenia may be related to mechanisms leading to HIT and more research is required to delineate this effect. Of note, although the rare documented cases of ON-related thrombocytopenia in humans have generally had a late onset [9], other features such as dose-dependency and lack of documented thrombosis do not seem to match the HIT model.

While the intrinsic molecular properties of ONs are an important risk factor for thrombocytopenia that can be addressed to a certain extent at the design and screening stages, a recent review of available drug development data [38] clearly suggests a higher risk for thrombocytopenia with increased exposure. Platelet reductions occurred in humans with doses higher than 175 mg/week and for three out of sixteen MOE-ONs incidence in platelet reductions at doses >175–275 mg/week were 20%, rising to as much as 50% at doses of >475 mg/week. This is in line with earlier observations that high peak plasma concentrations (C_max_) of ONs might be an important contributing factor. For example, a second-generation ON drug candidate, Volanesorsen, targeting Apolipoprotein C-III, which has been tested in a phase I clinical study in healthy subjects, showed a non-linear (4.5 fold) increase in exposure when doubling the dose from 200 mg to 400 mg per week, resulting in severe thrombocytopenia in an undisclosed number of patients at 300 mg/wk (IONIS press release; [39]). Peak plasma concentrations of ONs at high doses have been reported to be in the single digit μM range and are thus in the same range as concentrations used in the current study evaluating potential thrombocytopenia risk factors. Counteracting this risk, the higher potency of LNA- and cET-modified ON drugs allows lower doses, resulting in lower plasma concentrations [40]. Further, the use of conjugated ONs such as GalNAc-targeting to the liver has shown potential for even more significant dose reduction [40] and is therefore expected to lower peak plasma exposures substantially below the risk thresholds.

The use of animal models was out of the scope of this work. The reported ON-related thrombocytopeniae are rare events where the individual patient phenotype may be at play [38] and although animal models may shed further light on some mechanistic pathways, their clinical predictive value is unclear [13].

In the present study the PS content of an oligonucleotide has been identified as a major contributor for ON-mediated platelet binding and activation. In addition, we could demonstrate that these effects can be dramatically reduced by the incorporation of LNAs into the wings of PS-ONs. Moreover, due to higher hybridization efficiency of LNA-modified ONs, shorter lengths are adequate for high affinity binding to RNA targets (e.g. 16–14’mers), which in turn reduces PS content. The reduced PS content of shorter ONs thus should also reduce the risk of platelet binding and activation and probably also thrombocytopenic risk. Overall, both our own data and publically available data suggest that diverse risk factors arising from (a) ON molecular properties, (b) dose levels, as well as (c) patient populations (disease context) are of relevance for thrombocytopenic events observed clinically. The work presented here provides a means to address some of the risk factors originating from the nature of the drug molecule early on at design and screening stage. We have implemented *in vitro* cellular tools that allow assessing the impact of introducing sequence modifications in ONs, such as LNAs, which may lower the thrombocytopenic risk. The factors discussed above may only partially cover the pathways at play. Nevertheless, the ease of implementation of the corresponding assays supports their balanced use in discovery screening to deprioritize compounds bearing a higher risk level.

## Supporting information

S1 File Raw data figures. (XLSX)
